# A hybrid CNN-spectral architecture for non-contact respiratory rate estimation using multi-region optical-flow analysis

**DOI:** 10.1371/journal.pone.0325340

**Published:** 2026-02-20

**Authors:** Sreya Deb Srestha, Sungho Kim

**Affiliations:** Department of Electronic Engineering, Yeungnam University, Gyeongsan, South Korea; Ramaiah Institute of Technology, INDIA

## Abstract

Respiratory rate (RR) is a key indicator for assessing health conditions, yet noncontact measurement remains challenging due to motion artifacts, lighting variability, and skin-tone differences. This study presents a robust framework combining a custom convolutional neural network (CNN) with spectral analysis of optical-flow signals to estimate RR accurately across diverse population. Respiration-induced motion is extracted from six anatomical regions: forehead, cheeks, upper chest, and shoulders. Adaptive Kalman filtering combined with signal-to-noise ratio (SNR)-based weighted fusion enables reliable RR estimation. To improve generalizability, extensive data augmentation was applied, simulating illumination conditions ranging from normal to low light. The experimental results indicate that the proposed method achieves a mean average error (MAE) of 0.61–0.95 breaths per minute (bpm) for different skin tones and ages, within the clinically relevant range. These findings support the effectiveness of the multi-region CNN-spectral framework as a reliable, noncontact, real-time respiratory monitoring solution with potential for continuous healthcare and telemedicine applications.

## 1 Introduction

RR is a vital physiological sign, as fluctuations in respiratory patterns often indicate the onset of serious health complications. Studies have shown that respiratory data can be used to predict conditions such as chronic heart failure, cardiopulmonary arrest, and pneumonia [1–3]. During COVID-19 pandemic, there was a direct relation between respiratory and health status, where early detection of irregularities of RR has been proved to be crucial for timely medical intervention. Traditionally, RR is measured using invasive, contact-based systems that require sensors to be directly attached to the body of the patient, causing discomfort and skin irritation [4]. Moreover, the systems can be impractical for continuous monitoring due to limited accessibility, poor portability, and the need for trained personnel to operate them. In contrast, noninvasive techniques offer several advantages, such as eliminating skin irritation caused by contact sensors, allowing for seamless integration into daily life, and serving as a reliable alternative to wearable sensors for long-term monitoring [5]. Consequently, contactless vital sign monitoring has emerged as a promising solution to overcome these limitations of contact-based systems. Remote photoplethysmography (rPPG) is a novel, noncontact method that utilizes consumer-grade cameras to measure various physiological signals, including heart rate and respiratory rate.

The rPPG method has been employed to monitor subtle color changes in the skin region due to light reflectance that correlate with pulse-related components due to cardiac activities—using a standard RGB camera [6–10]. While the potential of rPPG is promising, reliably extracting pulse rate from video remains challenging. Overall method’s accuracy can be affected by factors such as motion artifacts, variations in skin tone, and ambient lighting conditions [11]. Another limitation of rPPG method is the influence of nonrespiratory physiological processes on the signal [12].

To address these challenges, various rPPG-based methods have been deployed, leveraging different regions of interest (ROI) for vital sign estimation using conventional RGB cameras. For RR estimation, one approach analyzes edge changes in the shoulder area of the subject [13], while another fuses pixel motion and intensity signals from the shoulder region, incorporating motion signals [14]. Several studies have focused on the forehead or cheek regions to estimate RR during non-voluntary motion [15]. Other studies indicate that the nostril region can also provide reliable estimates when there is no voluntary movement [16]. However, single-ROI or single-modality methods often struggle under realistic conditions where demographic variation, involuntary motion, and inconsistent lighting can degrade signal quality.

To address the limitations of single-modality, this study proposes a hybrid framework that fuses deep learning and classical signal processing to yield robust, noncontact RR estimates across different skin tones and natural environmental scenarios. The architecture processes six anatomically informed ROIs and combines spatial and temporal motion features for improved resilience to artifacts and demographic variability.

The proposed method combines CNN with conventional signal processing techniques using an SNR-driven weighted fusion that adaptively scales each ROI’s contribution. Additionally, the system employs an advanced optical-flow algorithm tailored to optimize feature extraction for subtle respiratory movements. This combined design improves signal reliability across varied skin tones and environmental conditions. The key contributions of this study are as follows:

- **A multi-ROI deep-learning and spectral-analysis framework for respiratory signal extraction:** This study introduces a unified architecture that simultaneously processes six anatomically distinct ROIs—combining spatial with temporal patterns via CNN and spectral analysis. This data-driven fusion enhances signal robustness compared to single-ROI methods, reducing sensitivity to localized motion artifacts or occlusions.
- **A skin-tone-specific RR estimate approach:** By quantifying real-time signal quality using SNR and dynamically reweighting ROI contributions, our approach directly compensates for light-absorption differences across skin tones. This addresses a critical research gap in existing noncontact vital-sign monitors, ensuring consistent accuracy for diverse populations and laying groundwork for generalizing telemedicine deployments.
- **An adaptive optical-flow algorithm for detecting respiratory motion:** To capture subtle face and chest movements in complex scenarios such as minor subject motion and changeable lighting, we modify a multi-scale pyramid Farneback implementation which is enhanced with contrast limited adaptive histogram equalization technique (CLAHE) and robust outlier handling. This development greatly increases motion-feature reliability, supporting the framework’s precision across the tested conditions.

The remainder of this paper is organized as follows: Sect 2 provides a detailed review of relevant studies on remote respiratory monitoring, including optical-flow techniques, deep learning approaches, and the performance of existing systems. Sect 3 describes the experimental setup, data preprocessing techniques and proposed methodology, which integrates optical-flow analysis, custom CNN architecture. Furthermore, there is a detailed discussion about the multi-region tracking strategy and rationale behind it, along with an adaptive signal processing pipeline designed for consistent performance across subjects with diverse skin-tone variations. Sect 4 presents the experimental results, including performance metrics, comparisons across different skin tones, validation against ground truth measurements, and data augmentation findings. Sect 5 provides an in-depth analysis of the model’s performance, including visualizations, and Sect 6 discusses the result findings and limitations to outline future research directions. Finally, Sect 7 concludes the paper by summarizing the main contributions and highlighting the broader significance of the work.

## 2 Related work

Recent advancements in noncontact RR monitoring have utilized a wide range of sensor technologies, including acoustic sensing, temperature variation detection, motion tracking, and light-based sensing [17,18]. These techniques detect breathing-related movements in areas such as the upper chest, neck, and face using sensors like RGB cameras, depth imaging, and wireless sensors. In particular, optical flow-based tracking has been applied to estimate RR by monitoring specific anatomical landmarks [19–21]. Although RGB camera-based systems are capable of detecting subtle light intensity changes caused by chest motion, they are susceptible to the posture of the subject. For instance, standing postures produce less pronounced chest movements, making it difficult to achieve accurate estimation [21].

Several studies have proposed methodologies to improve contactless RR monitoring. Some approaches involve color space transformations to extract respiratory signals via imaging photoplethysmography (iPPG) [22]. In contrast, others have investigated weak, respiration-synchronized head movements resulting from mechanical coupling between the head and chest regions [23]. Machine learning techniques have also been introduced to identify intrinsic mode functions (IMFs) that automatically represent the breathing signals [24]. These methods often employ face detection tools-such as the Haar cascade classifier, similar to the Viola-Jones algorithm [25] to define a forehead-centered ROI for signal extraction. Additionally, further studies have explored breathing-induced movements in the chest and abdomen region [26], as well as the use of head motion to detect artifacts in rPPG systems under synchronized and controlled breathing conditions [27]. Few studies have also investigated optimal combinations of RGB channels, with hemispherical surface grid search results indicating that the green channel is the most effective for baseline modulation [28].

Breathing activity can be detected through various mechanisms, including plethysmographic modulation [29], airway temperature fluctuations [30], and external respiratory sounds in the surrounding environment [31]. However, respiration-induced movement—which is not visible to the naked eye—remains one of the most direct and reliable indicators of respiratory activity. Accordingly, facial movements, especially in the nostril region, have been extensively studied [16,32]. Moreover, some studies have evaluated RR using only head movement, observing that even minimal head motion is modulated by the respiratory cycle [4]. The key challenge faced by all motion-based methods is differentiating between respiration-related motion and motion from other sources, an issue that becomes increasingly complex in real-world conditions.

Conventional RGB camera-based RR estimation methods typically extract respiratory signals from the facial region using blood volume pulse signals, or by observing breathing-induced motion in the chest, shoulders, and abdomen. However, deep learning-based approaches for RR estimation have received comparatively less attention than their widespread applications in heart rate estimation through rPPG and RGB video analysis. Recent studies have incorporated deep learning approaches for RR estimation. Proposed methods include CNN-based models for skin segmentation to extract RR from visible skin areas [33], as well as techniques to combine CNN-based ROI detection with clustering methods to isolate respiration-relevant pixels [34]. Researchers have also implemented different deep neural networks such as CNN models for rPPG signal extraction and spatiotemporal representation, enabling the estimation of vital signs from facial video sequences recorded after physical activities [35]. Furthermore, spatial-temporal convolution-based models have been identified to exploit the temporal information present in facial videos for rPPG signal estimation [36,37].

Despite these advancements, existing methods show several limitations. As noted earlier, single-ROI approaches remain vulnerable to posture changes, partial occlusions, and localized artifacts [21,38,39]. Furthermore, skin tone variation is an overlooked aspect that can significantly affect the quality of the extracted signal, resulting in variability in performance across demographic groups [40–42]. Additionally, separating breathing-induced motion from noisy signals remains challenging under realistic conditions [39,43]. Finally, while deep learning algorithms have shown promising results in related areas such as heart rate estimation [44], its integration with conventional signal processing techniques for reliable RR estimation so far has been limited, preventing existing methods from fully leveraging the complementary strengths of data-driven techniques [45,46].

Given these challenges, deep learning offers a promising direction for RR estimation. Among deep learning approaches for biosignal analysis, 1D CNNs offer distinct advantages over recurrent and transformer-based architectures. Convolutional filters effectively capture local temporal dependencies in respiratory signals [47], while enabling parallelizable computation essential for real-time monitoring unlike LSTMs and GRUs [48]. Additionally, 1D CNNs require fewer parameters than transformer models, which typically exceed tens of millions [49], making them well-suited for limited training data [50]. Prior studies demonstrate comparable or superior performance to recurrent networks for periodic biosignal analysis [44,45], and techniques such as batch normalization and dropout further enhance generalization [51,52].

Building on these advantages, an integrated framework combining deep learning with traditional signal processing is introduced within a unified multi-region architecture. Respiratory-related motion is simultaneously extracted from six anatomically defined ROIs, reducing reliance on any single region and enhancing resistance to motion artifacts. An adaptive signal-quality assessment module dynamically weights each ROI based on real-time SNR estimation, mitigating skin-tone-related signal variability. Furthermore, the incorporation of optical-flow–based motion tracking into a custom temporal CNN enables effective separation of respiration-induced motion from noise. By jointly leveraging spectral analysis and learned temporal features, the framework ensures consistent RR estimation across subjects with varying skin tones and environmental conditions.

## 3 Materials and methods

**Fig 1:**
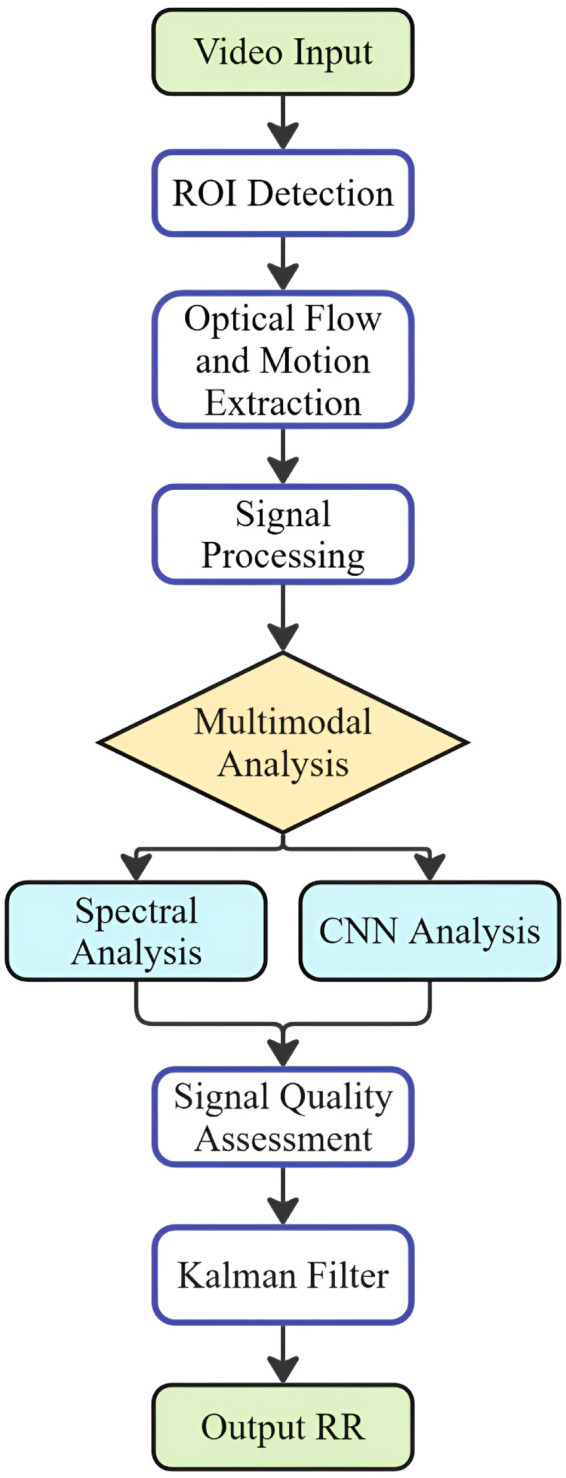
Overview of the proposed multimodal RR estimation methodology. This flow diagram illustrates how raw video input is processed and fused through signal analysis and a custom 1D CNN framework to produce robust RR estimates.

The proposed system architecture for RR monitoring consists of four primary components: (1) multi-region motion detection via the advanced Farneback optical flow algorithm with CLAHE; (2) a feature extraction module employing band-pass filtering (0.1–0.8 Hz) and Savitzky–Golay smoothing, along with time-domain and frequency-domain feature extraction; (3) a custom 1D CNN model that incorporates these six-channel feature sequences and spectral analysis to predict initial RR estimates; and (4) adaptive fusion and Kalman filtering, which weight and combine CNN and spectral outputs from each ROI with an improved SNR value for a real-time RR measurement. Overall workflow of the proposed methodology is illustrated in Fig 1. Each component will be described in the following sections.

### 3.1 Experimental setup and data collection

**Fig 2:**
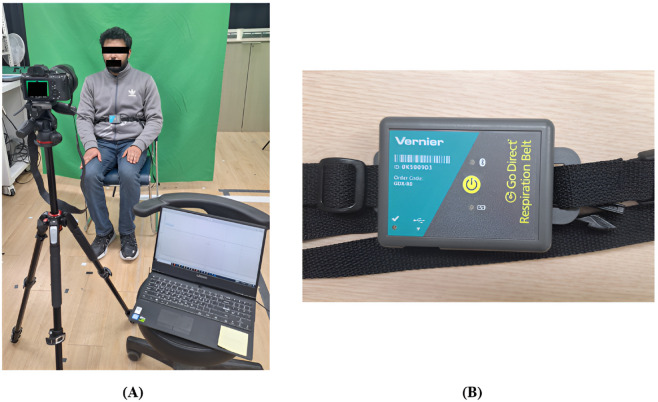
Experimental setup and ground truth measurement. **(A)** The subject was seated in front of an RGB camera at a 1-meter distance while wearing the go direct respiration belt. **(B)** Close-up view of the respiration belt used for contact-based measurement.

Experimental data were collected using a Sony Alpha 7 III camera, which recorded videos at a resolution of 6000 x 4000 pixels. The camera was positioned at a fixed distance of 1 meter from the subjects (Fig 2A) to ensure consistency in framing and image quality. The recording captured the face and upper chest of each subject and included respiration-induced movements. Video sequences were recorded at 30 frames per second (fps) under ambient lighting conditions, including both shaded and illuminated regions.

Lighting conditions were standardized using ambient indoor illumination measured at approximately 500 lux with a calibrated lux meter positioned at subject level. While this represents typical indoor lighting rather than clinical-grade controlled illumination, it was chosen specifically to reflect realistic deployment scenarios in home healthcare and telemedicine settings.

A green curtain was used as the background, while all other environmental factors remained natural and unchanged. The participants were instructed to remain seated and breathe naturally. No additional measures were taken to minimize noise or movement disturbances during the recording process.

Participants completed a brief survey documenting the current status of their health condition. No restrictions were imposed on previous activities, and participants rested approximately 2–3 minutes before measurement. This protocol reflects realistic, unconstrained monitoring conditions rather than controlled settings.

The dataset comprises recordings of 28 participants (14 male, 14 female) from South Asian, Middle Eastern, and East Asian regions, with ages ranging from 25 to 62 years. Skin tone was categorized into five levels inspired by the Fitzpatrick skin type scale (I–V). Classification was done through visual assessment conducted under controlled lighting conditions by observing exposed facial and forearm skin regions. This categorization represents a subjective grouping performed for analytical purposes rather than a clinical dermatological classification. For analysis, subjects were further categorized into subgroups as lighter skin tones (I–II) and darker skin tones (III–V), consistent with prior non-contact physiological monitoring studies [53].

A summary of the data collection setup and subject characteristics is provided in Table 1. All participants provided written consent via detailed consent forms, and the experiment was conducted in 2 days, on 11 February and 21 July 2025. The study received ethical approval from the Yeungnam University Institutional Review Board (Approval number: 7002016-A-2024-114), and the collected data were accessed and analyzed from 11 February through December 2025 to develop and validate the proposed algorithm. Each recording session lasted 60 seconds. Ground truth RR was simultaneously collected using a contact-based sensor, the go direct respiration belt (Code: GDX-RB) (Fig 2B). The ground truth data were recorded in CSV format for subsequent result analysis and comparing ground-truth value to validate the model’s performance metrics.

**Table 1:** Summary of the custom dataset and data acquisition protocol.

Parameter	Description
Camera model	Sony Alpha 7 III
Video resolution	6000 × 4000 pixels
Frame rate	30 fps
Camera distance	1 meter from subject
Lighting condition	Ambient indoor lighting (approximately 500 lux), with shaded and illuminated areas
Background	Green curtain backdrop
Recording duration	60 seconds per subject
Participants	28 total (14 male, 14 female)
Ethnicity	South Asian, Middle East and East Asian
Age range	25–62 years
Skin tone variation	Diverse (Fitzpatrick-inspired visual scale types I-V)
Ground truth sensor	Go Direct Respiration Belt (Code: GDX-RB)
Ground truth format	CSV data synchronized with each video timestamps

Table 1 summarizes the recording setup, subject demographics, and acquisition conditions used for the custom dataset.

The Go Direct Respiration Belt used for ground truth measurement has a manufacturer-specified accuracy of ±1 breath per minute within the 0–50 bpm range, with a resolution of 0.1 bpm [54]. The sensor operates via a force-sensitive resistor that detects thoracic expansion during breathing, providing a direct mechanical measurement of respiratory motion independent of optical properties or skin pigmentation [55]. This makes it suitable as a reference standard for evaluating camera-based respiratory estimation across diverse skin tones.

### 3.2 Pre-processing

The video input was initially standardized to ensure consistency and enhance quality across all recordings. Previous studies [56] have shown that higher resolution improves the accuracy of RR estimation; therefore, high pixel density was maintained to ensure reliable measurements under varying lighting conditions. Each frame is converted into a floating-point representation, with pixel values normalized to the range [0, 1]. To enhance visual detail—particularly in low-light or high-melanin contexts—an adaptive gamma correction is applied, where the *γ* value is dynamically adjusted based on the frame’s luminance characteristics using the following equation:

$$\gamma =\frac{a}{L_{\text{avg}}+\epsilon },$$ (1)

where $L_{\text{avg}}$ is the average luminance of the frame, *a* is a scaling constant, and *ε* is a small positive value to avoid division by zero. It ensures that darker frames (lower $L_{\text{avg}}$) are enhanced with higher gamma values, thereby improving contrast and detail visibility in low-light scenarios as well as on high melanin-induced skin variations.

After frame normalization, a frame-quality assessment module evaluates each frame for motion blur, exposure, and improper exposure, adaptively rejecting or reweighting frames that fail to meet quality thresholds to mitigate skin-tone-dependent errors.

Next, a Haar feature-based cascade classifier is used to detect the face of the subject. After the face from the frame has been detected, six key ROIs are dynamically identified as the forehead, left and right cheeks, upper chest, and left and right shoulders. The system continuously adjusts the positions and size of the ROIs to monitor subject movement and individual variations in facial and body structure. Real-time ROI tracking mechanism maintains ROI stability while adapting to positional changes. These regions are selected due to their physiological relevance in capturing respiration-induced motion. To further enhance feature visibility, each ROI undergoes the CLAHE technique [57], which increases local contrast and highlights subtle details without amplifying noise. Gaussian smoothing combined with bilateral filtering suppresses high-frequency noise while preserving critical edge information. This two-stage filtering process retains respiratory motion while reducing noise artifacts. The adaptive preprocessing pipeline compensates for variations in skin pigmentation and lighting conditions before motion extraction, thereby reducing estimation bias and optimizing RR estimation performance.

**Fig 3:**
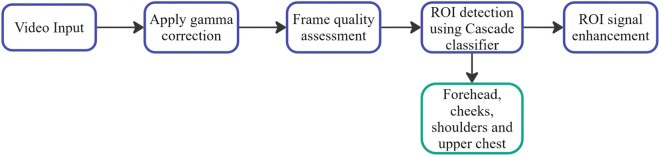
Pre-processing of input video. The input video undergoes multiple stages to extract ROI and produce a standardized signal.

Fig 3 illustrates the complete preprocessing pipeline, detailing the sequential steps from video input standardization to ROI detection and signal enhancement workflow.

### 3.3 ROI selection and optical flow-based respiration signal extraction

The selection of the six ROIs is based on physiological, biomechanical, and technical considerations to ensure comprehensive coverage and redundancy for RR estimation. Physically, the upper chest region is chosen due to its close association with thoracic breathing; respiratory-induced motion is most prominently visible in this area [23]. The facial regions, particularly the forehead, are relatively stable and largely free from significant muscular interference, making them ideal as a baseline for RR detection. The cheek region offers complementary cues through color variation and subtle motion, which indicates blood volume changes related to the breathing cycle. The shoulder regions capture auxiliary respiratory movements, especially during deeper or more forceful breaths, and thus provide additional validation.

These ROIs are automatically positioned in relation to the detected face bounding box, allowing the capture of diverse breathing motions without requiring manual intervention. If the face detector produces a rectangle (*x*,*y*,*w*,*h*), each ROI *i* is defined as:

$${ROI}_{i}=(x+\alpha_{i}\;w,\;y+\beta_{i}\;h,\;\gamma_{i}\;w,\;\delta_{i}\;h),$$ (2)

where $\alpha_{i}$ and $\beta_{i}$ represent the normalized horizontal and vertical offsets from the top-left corner of the face, while $\gamma_{i}$ and $\delta_{i}$ specify the width and height of the ROI to establish the position and size of each region, including the forehead, cheeks, upper chest, and shoulders. This unified formulation ensures that all ROIs automatically adapt to the subject’s face dimensions and alignment, covering both primary thoracic expansion and auxiliary facial or shoulder movements. Each ROI is then clamped to the image boundaries before extracting its raw and filtered signals using band-pass filtering.

**Fig 4:**
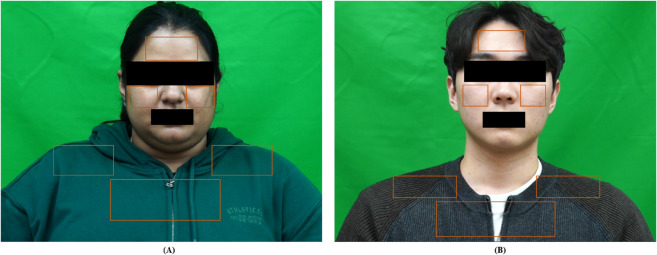
Illustration of automatic ROI detection on two subjects with different skin tones: (A) darker and (B) lighter. Both images show how the six rectangular ROIs—forehead, cheeks, upper chest, and shoulders—dynamically adapt to individual facial features and hair coverage, ensuring consistent respiratory signal capture.

Fig 4 presents two example cases: one participant with higher skin pigmentation (Fig 4A) and another with lower skin pigmentation (Fig 4B). In both cases, despite variations in facial structure, hairline, and lighting, the algorithm consistently positions stable forehead, cheek, chest, and shoulder ROIs, confirming both its anatomical adaptability and its reliability for real-time, multiregion respiratory monitoring.

To validate the robustness of our automatic ROI placement algorithm, we compared each detected rectangle to manually annotated ground truth values on each ROI of the overall video frames. Then we computed the dice coefficient using the following equation:

$$Dice\;=\;\frac{2\;|\text{detected}\;\cap \;\text{ground truth}|}{\left|\text{detected}|\;+\;|\text{ground truth}\right|}$$ (3)

For each region in every frame, Table 2 summarizes the per-ROI statistics and overall reliability. The mean dice coefficients range from 0.978 to 0.986 with standard deviations between 0.008 and 0.016, and all regions achieve a minimum score of 0.891. The overall mean dice score across all ROIs is 0.983±0.013, indicating that all frames exceed a dice score of 0.70, which confirms consistently accurate localization.

**Table 2:** Per-ROI dice coefficient statistics.

Region	Mean Value	Standard Deviation (STD)	Min value	Max Value
Forehead	0.986	0.008	0.905	1.000
Cheek (left)	0.984	0.009	0.913	1.000
Cheek (right)	0.984	0.009	0.908	1.000
Chest (upper)	0.985	0.011	0.925	1.000
Shoulder (left)	0.979	0.016	0.896	1.000
Shoulder (right)	0.978	0.016	0.891	1.000
**Overall**	**0.983**	**0.013**	**Dice Score > 0.70**	

**Fig 5:**
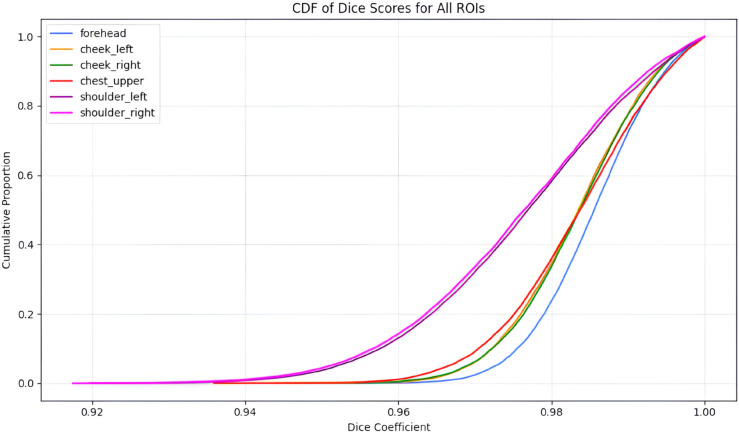
CDF of ROI placement accuracy. The CDF of dice coefficients for the six automatically placed ROIs demonstrates consistently high overlap with ground truth across all frames.

The cumulative distribution function (CDF) of estimated dice scores for each of the six ROIs is plotted in Fig 5, which also shows that more than 80% of ROI placements achieve an optimal coefficient value over 0.98, indicating a consistently high overlap with ground truth.

**Fig 6:**
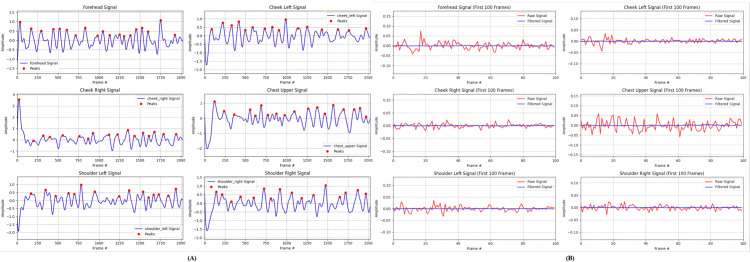
Peak detection and signal comparison for the six ROIs. (A) Peak detection results for each ROI. (B) Comparison of raw and filtered signal amplitudes for each ROI.

Fig 6A illustrates the peak detection results across all six ROIs over the full recording duration. The detected peaks (red markers) correspond to respiratory cycles, with the chest ROI exhibiting the most pronounced and consistent periodicity due to direct thoracic expansion. The forehead and cheek signals show lower amplitude peaks, while the shoulder regions capture secondary respiratory motion with moderate peak prominence. In Fig 6B, the raw signals (red traces) from each ROI are plotted over time. These signals vary in amplitude and noise level, reflecting the distinct biomechanical and physiological characteristics of each region.

Notably, the cheek ROIs exhibit higher noise levels due to lighting-induced artifacts from their facial curvature, which creates shadows and specular reflections under non-uniform illumination. The SNR-based weighted fusion mitigates this by reducing cheek signal contributions when the signal quality degrades. Comparing raw and filtered signals confirms that preprocessing preserves the respiratory component while reducing noise. This validates the multi-ROI approach, where complementary information from multiple regions ensures reliable tracking even when individual ROIs are affected by occlusions or artifacts.

Following ROI extraction and preprocessing, dense optical flow is computed between successive video frames using the Farneback algorithm. The algorithm estimates pixel-wise motion and returns a two-dimensional flow field defined as:

F(x,y)=(u(x,y),v(x,y)) (4)

where u(x,y) and v(x,y) represent the horizontal and vertical motion components, respectively, at the pixel location (x,y). The motion magnitude M(x,y) is calculated as the Euclidean norm of these components:

$$M(x,y)=\sqrt{u(x,y{)}^{2}+v(x,y{)}^{2}}$$ (5)

To justify the selection of the optical flow algorithm used in the framework, a subject-level comparison was conducted among four representative approaches, such as Lucas–Kanade, TV-L1, Deep Flow, and the Farneback method. For each subject, motion signals were independently extracted using each optical flow technique, and the SNR was computed by aggregating temporal measurements across the entire session. This ensured that each subject contributed a single representative value per method, thereby preventing frame-level bias and data leakage.

**Fig 7:**
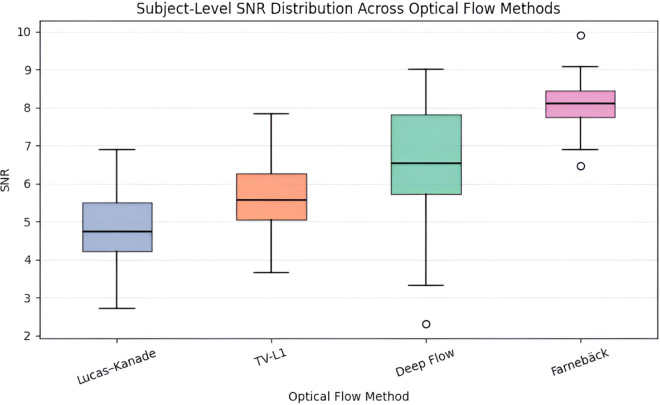
Subject-level comparison of motion signal quality across different optical flow methods for respiratory motion extraction. Each distribution summarizes subject-wise quality scores, where higher median and lower dispersion indicate more stable respiratory motion estimation.

Fig 7 illustrates the distribution of subject-wise SNR values across the four methods. While all approaches exhibit overlapping performance metrics, the Farneback method consistently demonstrates a higher median SNR and a more compact interquartile range, indicating improved stability and consistency across subjects. In contrast, Lucas–Kanade and TV-L1 show greater variability, reflecting sensitivity to noise and subtle non-respiratory motion, while Deep Flow presents higher dispersion despite occasional strong responses. These findings suggest that although alternative optical flow techniques may perform well for specific subjects or conditions, the Farneback method offers superior overall consistency when evaluated at the subject level.

Optical flow is employed to capture subtle pixel-level motion associated with respiratory activity. By measuring the motion magnitude, the system can effectively distinguish between respiratory-related movements in the head and upper chest and other unrelated motion artifacts. This distinction is critical for enabling the integration of motion-based signals with color-based rPPG signals.

The motion amplitude is smoothed using a Gaussian filter to reduce noise and enhance signal reliability. The resulting signal is then normalized via mean subtraction and division by the standard deviation. To further refine the signal, outliers are then removed using the interquartile range (IQR) method: the 20th percentile (Q1) and 80th percentile (Q3) are computed, and any values outside this range are discarded.

$$\left[Q_{1}-1.5\;\text{IQR},\;Q_{3}+1.5\;\text{IQR}\right]$$ (6)

The normalized mean motion was further refined using a sigmoid-like function, which is defined as:

$$\text{Motion Score}=\frac{2}{1+e^{-N}}-1$$ (7)

$$N=\frac{\text{mean}}{\text{std}+\epsilon }$$ (8)

where *N* represents the normalized motion intensity, calculated as the ratio of the mean to the standard deviation of the motion signal-with *ε* added to prevent division by zero. This transformation compresses the dynamic range of motion intensity values by mapping them onto a bounded interval. It mitigates the influence of outliers caused by noise while preserving subtle respiratory movements. As a result, the motion score offers a stable and consistent metric that can be reliably used in analysis stages.

### 3.4 Signal processing pipeline

The methodology incorporated a comprehensive signal processing pipeline to extract and enhance respiratory signals from the motion data. In the initial preprocessing stage, temporal smoothing is applied using the Savitzky–Golay filter with a window length of 5. This filter was selected for its ability to reduce high-frequency noise while preserving the underlying characteristics of the respiratory signal. Following smoothing, the direct current (DC) component is removed from each signal through mean subtraction, which is expressed as:

$$s_{\text{dc}}(n)=s(n)-\frac{1}{N}\sum_{n=1}^{N}s(n)$$ (9)

where s(n) is the original signal and N denotes the total number of samples. This step eliminates baseline drift and centers the signal at zero. Next, the signal is normalized to have unit variance using the following transformation:

$$s_{\text{norm}}(n)=\frac{s_{\text{dc}}(n)}{\sigma_{s}+\epsilon }$$ (10)

where $\sigma_{s}$ denotes the standard deviation of the signal and *ε* is a small constant added to ensure numerical stability.

To isolate spectral features of the respiratory signal, two-stage bandpass filtering is employed. The first stage uses a second-order Butterworth filter with the following transfer function:

$$H(s)=\frac{1}{\sqrt{1+{\left(\frac{\omega }{\omega_{c}}\right)}^{2n}}}$$ (11)

where $\omega_{c}$ is the cutoff frequency and *n* denotes the filter order.

Zero-phase filtering is implemented using forward-backward filtering to preserve the temporal structure of the signal. Maintaining phase integrity is critical for accurate frequency estimation and reliable RR detection. This combination of smoothing, normalization, and spectral filtering isolates the respiratory component of the signal while removing high-frequency noise and low-frequency drift, yielding a clean signal suitable for further analysis.

The parameters were selected empirically based on the experimental observations and prior studies. For instance, the Savitzky–Golay filter is configured with a window length of 5 and a polynomial order of 2 to smooth the signal while preserving critical features effectively. The second-order Butterworth filter is chosen for its flat passband response, and its cutoff frequencies are aligned with the typical respiratory frequency range to ensure effective suppression of high-frequency noise and low-frequency drifts. Overall, the sequential integration of temporal smoothing, baseline correction, normalization, bandpass filtering, and outlier removal ensures that the final respiratory signal retains pulse-related components. This optimization minimizes noise and artifacts, preparing the signal for subsequent analysis.

### 3.5 Spectral and time-domain approaches for RR estimation

Spectral and time-domain approaches are employed to estimate RR, leveraging the distinct characteristics of the processed signals and ground truth data. These techniques enhance robustness by providing independent techniques that can be cross-validated and fused.

For frequency-domain analysis, Welch’s method [58] is used to estimate the power spectral density (PSD) of the respiratory signal. This method applies a Hann window and uses adaptive segment lengths of up to 4 seconds with a 75% overlap across segments. The PSD is computed by averaging the periodograms of each segment, as described by

$$P_{xx}(f)=\frac{1}{L}\sum_{l=1}^{L}|X_{l}(f){|}^{2}$$ (12)

where *L* is the number of segments, and *X*_*l*_(*f*) is the Fourier transform of the *l*-th segment.

Peak detection is then carried out within the physiologically relevant frequency range of 0.15-0.5 Hz. Peaks are identified based on standard criteria: prominence, width, and minimum distance. The RR in bpm is computed as:

$$\text{bpm}=60\times f_{\text{peak}}$$ (13)

Two time-domain techniques are also used to estimate RR:

- Zero-Crossing Rate Method: This approach counts the number of times the signal crosses zero and computes the interval between crossings. The RR is then estimated as:
  $$\text{bpm}\approx \frac{\text{fps}\times 30}{\Delta n}$$ (14)
  where, fps is the sampling rate and Δ*n* denotes the mean interval between zero crossings.
- Peak-Based Rate Method: This method calculates the average interval between successive peaks ($\Delta n_{\text{peak}}$) and estimates the RR as
  $$\text{BPM}\approx \frac{\text{fps}\times 60}{2\Delta n_{\text{peak}}}$$ (15)

The multiplication factor of 2 accounts for the bidirectional nature of respiratory cycles (i.e., one full breath includes inhalation and exhalation). All estimation methods are constrained to output values within the clinically valid range of 9 to 30 bpm to ensure physiological plausibility.

Spectral and time-domain methods offer distinct but complementary advantages. The spectral approach excels at isolating dominant frequency components in relatively denoised signals, while the time-domain methods provide accurate estimates even in the presence of transient artifacts and irregular breathing patterns. Hyperparameter selection, such as a 4-second window with 75% overlaps while using Welch’s method, and thresholds for peak detections were optimized to balance pixel resolution and signal stability. Additionally, the zero-crossing and peak-based methods exhibit strong resilience to amplitude fluctuations, which enhances their reliability under varying noise conditions. Spectral estimation outputs are subsequently combined using a weighted fusion strategy guided by signal quality metrics. This fusion ensures that the final RR remains within physiologically plausible limits.

### 3.6 CNN-based estimation and data fusion

**Fig 8:**
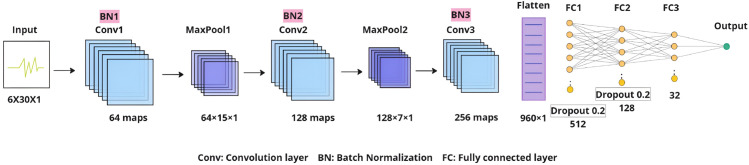
Proposed CNN architecture for RR estimation. The CNN architecture processes multichannel input sequences to extract respiratory-related features, using convolutional layers for temporal feature extraction and fully connected layers for final prediction.

In parallel with the traditional signal processing methods, we developed a custom CNN architecture, which is integrated into the pipeline to learn complex temporal patterns from the multichannel respiratory signals extracted from different ROIs, as illustrated in Fig 8.

The network processes input sequences in the time domain. Each input layer has shape (*B*,6,*L*), where *B* is the batch size, 6 is the number of tracked ROIs, and *L* is the fixed window length in frames. The time-domain input consists of raw frame-to-frame optical flow motion signals extracted from each ROI, including pixel displacement magnitudes and average motion vectors representing subtle respiration-induced movements.

The CNN architecture is designed to extract stronger motion features progressively by combining three one-dimensional convolutional layers with batch normalization, ReLU activations, and max pooling. Each convolutional layer performs a temporal filtering operation on the input sequence defined as:

$$y[i]=\sum_{j=-2}^{2}x[i+j]\cdot w[j].$$ (16)

where *y*[*i*] is the output at the time step *i*, and *w*[*j*] denotes the filter weights for a kernel to preserve the temporal resolution. Batch normalization and ReLU activation follow each convolution to stabilize training and introduce nonlinearity. Max pooling reduces the temporal dimension by half per layer, gradually focusing on the most salient motion features.

The output feature map is flattened and passed through three fully connected (FC) layers with ReLU and dropout regularization. The final FC layer maps the extracted features to a single continuous respiratory rate prediction. The complete network structure is summarized in Table 3.

**Table 3:** Proposed CNN network architecture for RR estimation.

Layer	Operation	Feature Maps	Input Size	Output Size
1	Conv1d(6–64, k=5, p=2) + BN + ReLU + MaxPool(2)	64	6×30	64×15
2	Conv1d(64–128, k=5, p=2) + BN + ReLU + MaxPool(2)	128	64×15/2	128×7
3	Conv1d(128–256, k=3, p=1) + BN + ReLU + MaxPool(2)	256	128×7	256×3
4	Flatten + Linear(768–512) + ReLU + Dropout(0.2)	512	768	512
5	Linear(512–128) + ReLU + Dropout(0.2)	128	512	128
6	Linear(128–32) + ReLU	32	128	32
7	Linear(32–1)	1	32	1

For training, a fixed sliding window is applied to the continuous ROI motion traces, where each sample is paired with the corresponding reference RR label at the window’s final frame.

To ensure reproducibility and subject-independent evaluation, model performance was assessed using subject-level *k*-fold cross-validation. In each fold, subjects were partitioned into mutually exclusive training, validation, and testing sets, with recordings from the same individual strictly confined to a single split to prevent data leakage. Approximately 70% of subjects were used for training, 10% for validation selected from the training pool, and the remaining 20% were reserved for testing within each fold. This evaluation protocol ensures that generalization is assessed across unseen individuals rather than across frames or temporal windows. Although the dataset includes a limited number of participants, the subject-level cross-validation strategy reduces optimistic bias and enables reliable within-dataset evaluation across skin-tone subgroups.

The network is trained for 200 epochs using the AdamW optimizer with a fixed learning rate of 0.001 and weight decay regularization to mitigate overfitting. A learning rate scheduler automatically reduces the learning rate when the validation loss does not improve significantly over a predefined number of epochs, enabling finer adjustments to the model parameters and preventing stagnation in training. The Huber loss is employed as the regression objective to balance sensitivity and robustness against outliers, which is defined as:

$$L_{\delta }(a)=\left\{ \begin{array}{cc} \frac{1}{2}a^{2}, & \text{if }|a|\leq \delta ,\\ \delta \left(|a|-\frac{1}{2}\delta \right), & \text{otherwise}, \end{array}\right.$$ (17)

A summary of the proposed CNN framework training and hyperparameter settings is given in detail in Table 4.

**Table 4:** Training configuration and hyperparameters.

Parameter	Value
Evaluation Protocol	Subject-level *k*-fold cross-validation
Number of folds (*k*)	5
Data Partitioning	70% train, 10% validation, 20% test (per fold)
Optimizer	AdamW
Learning Rate	0.001
Epochs	200
Loss Function	Huber loss
Weight Decay	Enabled (AdamW)
Learning Rate Scheduler	ReduceLROnPlateau
Dropout	0.2 in FC layers

**Fig 9:**
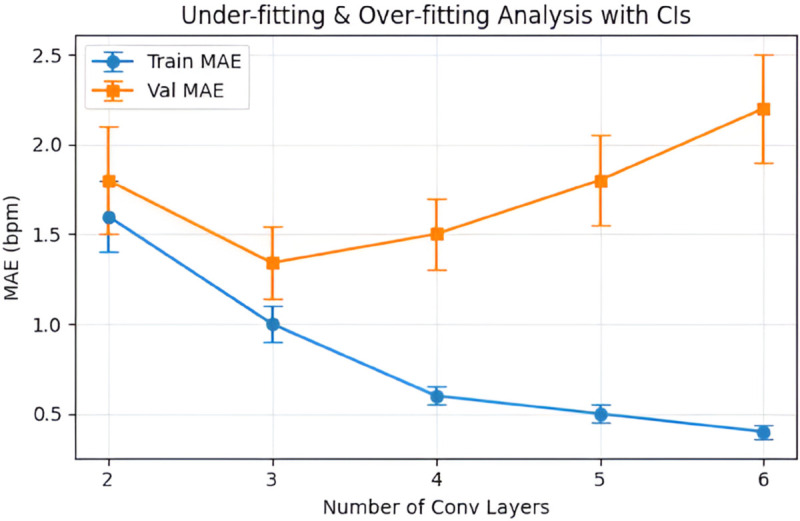
Under–fitting and over–fitting analysis based on the number of layers of the proposed CNN architecture. The plot shows the training and validation MAE as a function of the number of convolutional layers. Three layers provide the best balance between learning capacity and generalization, supporting the final model choice.

To further validate the choice of optimal network layers, Fig 9 presents an under and over-fitting analysis that plots the mean ± standard deviation (SD) and mean absolute error (MAE) of the training and validation as a function of the number of convolutional layers. With only two convolutional layers, the model clearly underfits the data, yielding high training (1.60 ± 0.20 bpm) and validation (1.80 ± 0.30 bpm) MAE values. Increasing to three layers—the final configuration adopted in this study—reduces the training MAE to 1.0 ± 0.10 bpm and the validation MAE to 1.34 ± 0.20 bpm. This corresponds to the observed mean performance on the testing dataset across different skin tones, as detailed in Sect 4. Adding further convolutional layers reduces the MAE during training but increases the validation MAE, indicating overfitting. These results confirm that the selected three-layer CNN achieves an effective balance between learning capacity and generalization for the motion signals.

The final RR estimation is obtained using the CNN-derived estimate with the spectral estimate. To determine the contribution of each ROI, an SNR-based signal quality measure is computed. The SNR for a given ROI is defined as:

$$\text{SNR}=10\log_{10}\left(\frac{\sigma_{\text{filtered}}^{2}}{\sigma_{\text{noise}}^{2}}\right)$$ (18)

After computing the SNR values for each ROI, they are averaged and normalized—then clipped to lie within the range [0,1]. Based on these normalized quality measures, a fusion weight *w* is assigned within the range [0.3, 0.7], balancing the contributions of estimation approaches.

$$R_{\text{final}}=w\cdot R_{\text{CNN}}+(1-w)\cdot R_{\text{spectral}}$$ (19)

This adaptive integration leverages the strengths of deep learning frameworks and classical spectral methods.

A Kalman filter is applied to refine the temporal sequence of predictions to improve the quality of RR estimates further. The Kalman filter, being recursive in nature, consists of a prediction step.

$$x_{k}=x_{k|k-1}+K_{k}(z_{k}-x_{k|k-1}),$$ (20)

where *K*_*k*_ is the Kalman gain, and *z*_*k*_ is the observed measurement obtained from the fusion of the CNN and spectral methods. A dynamic rate-limiting constraint is also implemented to prevent unrealistic or abrupt fluctuations in the estimated RR. These post-processing steps ensure the final output remains smooth, stable, and physiologically plausible—an essential requirement for real-time clinical and telehealth applications.

## 4 Model evaluation

In this section, we present a comprehensive evaluation of the proposed architecture using three primary datasets: (1) the publicly available PURE dataset (2) UBFC-rPPG dataset and (3) a custom dataset collected under varied skin tones and natural lighting conditions replicating real-world scenario. To validate proposed model performance, three primary metrics are used: MAE, root mean squared error (RMSE) and the Pearson correlation coefficient (*r*). MAE and RMSE quantify the magnitude of deviation between the estimated and ground truth RRs, while *r* measures the linear correlation between them. Here defined the following performance metrics equation:

$$\text{MAE}=\frac{1}{N}\sum_{i=1}^{N}|y_{i}-{\hat{y}}_{i}|,$$ (21)

$$\text{RMSE}=\sqrt{\frac{1}{N}\sum_{i=1}^{N}(y_{i}-{\hat{y}}_{i}{)}^{2}}.$$ (22)

$$r=\frac{\sum_{i=1}^{N}(y_{i}-\bar{y})({\hat{y}}_{i}-\bar{\hat{y}})}{\sqrt{\sum_{i=1}^{N}(y_{i}-\bar{y}{)}^{2}}\sqrt{\sum_{i=1}^{N}({\hat{y}}_{i}-\bar{\hat{y}}{)}^{2}}},$$ (23)

where ${\hat{y}}_{i}$ represents the estimated RR, *y*_*i*_ denotes the ground truth, $\bar{y}$ and $\bar{\hat{y}}$ are their respective mean values, and *N* is the total number of samples.

At first we evaluated the framework using the PURE dataset, which is recorded under controlled environmental conditions and used as a baseline for respiratory monitoring. As shown in Table 5, the proposed method achieved an MAE of 0.58 bpm and an RMSE of 0.78 bpm, demonstrating strong agreement with ground truth measurements. These results confirm the reliability in controlled scenarios.

**Table 5:** Ablation study of proposed method for RR estimation.

Dataset	Spectral MAE (bpm)	CNN MAE (bpm)	Proposed method MAE (bpm)	Proposed method RMSE (bpm)	Proposed method correlation coefficient (r)
PURE	1.03	0.95	0.58	0.78	0.88
UBFC-rPPG	1.05	0.95	0.60	0.81	0.87
Custom Intra-dataset (Light)	1.06	0.97	**0.61**	**0.78**	**0.87**
Custom Intra-dataset (Dark)	2.15	1.71	**0.95**	**1.08**	**0.75**
Custom Cross-dataset (Training on dark and testing on light)	2.48	1.83	1.37	2.20	0.63
Custom Cross-dataset (Training on light and testing on dark)	3.27	2.58	2.39	3.06	0.56

Table 5 reports the MAE and RMSE in bpm, along with the correlation coefficient (*r*) between the estimated and ground truth respiratory rates. The PURE and UBFC-rPPG dataset represents recordings under controlled conditions, whereas the custom dataset includes real-world recordings from subjects with varying skin tones.

To further assess the robustness of the method against moderate real-world variability, we applied our algorithm to the UBFC-rPPG dataset, which includes moderate illumination and motion variations. The architecture maintained high accuracy (MAE 0.60 bpm, RMSE 0.81 bpm), which was consistent and similar to the result findings on PURE demonstrating resilience to typical environmental noise and motion artifacts.

Furthermore, to compare, evaluation of the proposed model on the custom dataset—which comprises recordings under natural conditions and diverse skin tones—revealed subgroup differences. The lighter skin tone subgroup achieved an MAE of 0.61 bpm and an RMSE of 0.78 bpm, comparable to public dataset performance. In contrast, the subgroup with darker skin tones exhibited greater error, with an MAE of 0.95 bpm and an RMSE of 1.08 bpm.

To isolate each branch’s contribution across different skin-tone domains, a cross-dataset ablation study was conducted. The spectral-only analysis revealed limited transferability, with higher MAEs when parameters optimized for one skin-tone group were applied to another. Although the CNN-only approach generalized better, errors remained elevated when tested on opposite subgroups. The full CNN–spectral fusion consistently reduced cross-dataset errors, demonstrating the effectiveness of combining learned features with deterministic spectral analysis. The performance variations reflected the greater environmental complexity in the custom dataset, including uncontrolled lighting and skin reflectance differences, yet the proposed method maintained strong adaptability across both public and custom datasets.

**Fig 10:**
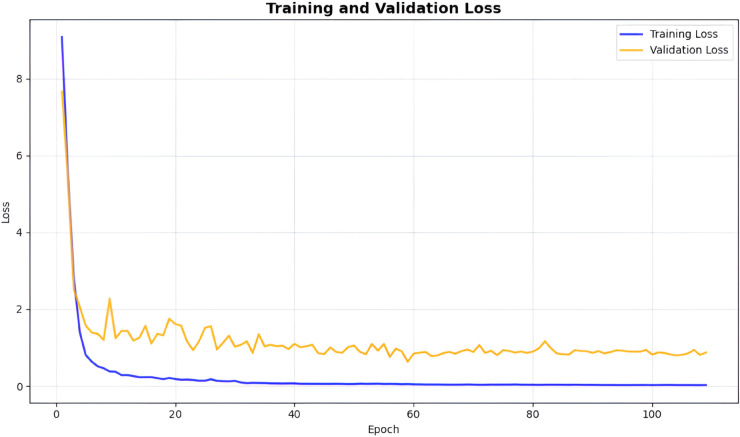
Training and validation loss curves. Losses were monitored over the first 110 epochs with early stopping, demonstrating smooth, parallel decline demonstrating stable convergence.

To verify training stability, both training and validation loss were monitored over 110 epochs (Fig 10). Training loss decreased from 0.3723 to 0.0250, indicating effective learning, while validation loss converged from 1.2451 to 0.8217 without divergence, confirming maintained generalization. This controlled reduction in both curves supported the reliability of the reported performance metrics.

Accurate ground truth measurement is critical for evaluating RR estimation methods, yet reference modalities vary considerably across datasets. Contact-based respiration belts, which measure thoracic or abdominal expansion, remain the most direct and widely used approach due to their sensitivity to actual respiratory motion [59]. In contrast, ECG-derived methods leveraging respiratory sinus arrhythmia (RSA) are fundamentally indirect and often less accurate. Recent studies explicitly treat ECG as a cardiac reference rather than a reliable respiratory ground truth [59,60].

**Fig 11:**
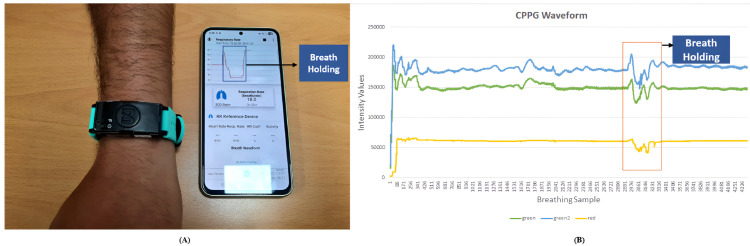
Visualization of PPG device and its raw signal output. (A) Subject wearing the contact PPG sensor, which streams physiological signals to a mobile app via Bluetooth. The app displays real-time pulse waveforms and heart rate in bpm. (B) Raw intensity waveforms from the PPG sensor, showing two green and one red channel. Despite breath-hold beginning near sample index 2941, the signal continues to exhibit amplitude fluctuations, indicating limited sensitivity to respiratory pauses.

Contact-based photoplethysmography (cPPG) is commonly used for ground truth RR measurement; however, its suitability for respiratory validation remains unclear. Fig 11(A) shows a subject wearing the MAXREFDES105# Health Sensor Band with real-time breathing rate displayed via Bluetooth connectivity, while Fig 11(B) illustrates the raw intensity traces from two green and one red channel. Notably, when the subject intentionally held their breath near sample index 2941, the waveform failed to reflect corresponding signal attenuation, instead exhibiting high-frequency jitter suggestive of motion artifacts or perfusion instability. This observation indicates that cPPG, while useful for cardiac monitoring, may lack sensitivity to respiration-induced variations during irregular breathing. Factors including motion artifacts, skin contact inconsistency, and peripheral vasoconstriction can compromise respiratory tracking reliability, challenging the assumption that cPPG provides an ideal respiratory reference.

Given these limitations, it is essential to distinguish between different contact-based ground truth modalities. Unlike cPPG, which relies on optical measurement of blood volume changes and is susceptible to skin pigmentation effects [41,42], the respiration belt used in this study operates via mechanical sensing of thoracic expansion. This mechanical approach provides a skin-tone-independent reference essential for evaluating camera-based methods across diverse populations [59,61,62].

Despite the availability of multiple reference modalities, no unified study has systematically compared respiration belts, ECG-derived systems, and cPPG under controlled conditions to establish a gold standard for RR monitoring. This absence of consensus complicates cross-study benchmarking and underscores the importance of dataset-specific context when interpreting results.

In addition to assessing the validity of contact-based references, a detailed comparison between the proposed framework and previously reported rPPG-based RR estimation techniques is presented in Table 6. Reference studies employed both public datasets—such as COHFACE [63] and AIR-125 [64]—and custom datasets targeting specific populations, including neonatal intensive care patients [65]. However, most prior methods did not account for skin tone diversity or lighting variability, factors that significantly affect signal quality in practical settings.

**Table 6:** Comparison of RR estimation methods using custom datasets.

Reference	Methodology	Dataset	MAE (BPM)
[35]	2D Spatiotemporal CNN	Custom	1.53
[66]	CNN architecture with MCV	Custom	1.7
[63]	Multitask Convolutional Attention Network with Pulse-Respiration Quotient	COHFACE	2.053
[67]	Eulerian magnification with a 3D CNN	Custom	2.29
[64]	Respiration flow-based network (AIRFlowNet)	AIR-125	2.91
[65]	Multitask CNN with blind source separation method	Custom	6.9
**Proposed**	**1D CNN–Spectral Architecture**	**Custom**	**0.61–0.95**

Comparison of MAE in bpm across various RR estimation methods on self-collected datasets, highlighting the competitive performance of the proposed 1D CNN–spectral hybrid architecture.

Since previous methodologies as well as this study were evaluated on independent datasets, it cannot be conclusively determined that performance would generalize equally across studies. Nevertheless, this comparative overview offers meaningful benchmarks and underlines the need for future studies to validate generalizability across diverse datasets and subject populations.

### 4.1 Data augmentation

Given the relatively small size of the custom dataset, we augmented each recorded video to simulate a range of illumination conditions and evaluate the algorithm’s robustness under illumination variation. The augmentation was performed by uniformly darkening each frame in the original video using a multiplicative brightness scaling approach. Specifically, for each pixel location (*x*,*y*) and RGB color channel *c*, the augmented pixel intensity was computed as:

$$I_{\text{aug}}(x,y,c)=\beta \cdot I_{\text{orig}}(x,y,c)$$ (24)

where $I_{\text{orig}}(x,y,c)$ is the original pixel intensity, $I_{\text{aug}}(x,y,c)$ is the darkened output, and *β* is the brightness factor ranging from 0 (completely black) to 1 (original brightness). The brightness factor *β* is derived from the desired darkness level D% as follows:

$$\beta =1-\frac{D}{100}$$ (25)

For example, a darkness level of 50% corresponds to β=0.5, meaning all pixel intensities are halved. Using this equation, three augmented versions of each original video were generated at D={25,50,75}%, in addition to the baseline (D=0%). This resulted in a total of 84 videos, maintaining consistent motion, skin tone, and background while varying only illumination. Such controlled augmentation procedure allows us to observe how progressive reductions in illumination affect the performance metrics of respiratory rate estimation.

**Fig 12:**
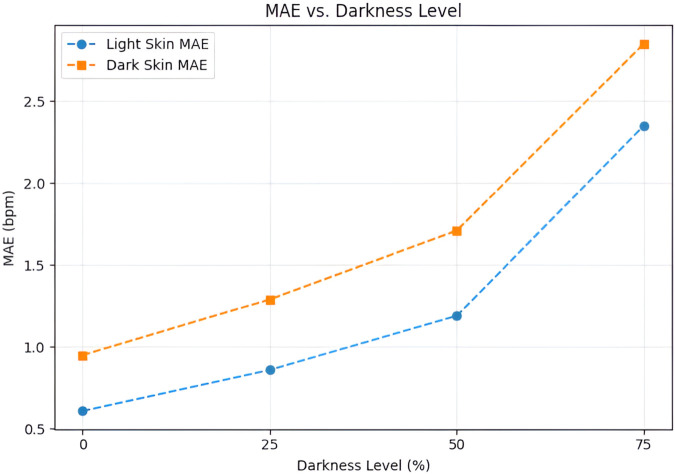
Effect of illumination reduction on MAE. Respiratory-rate estimation error is plotted against four levels of simulated darkness (0%, 25%, 50%, 75%) for light-skin and dark-skin subsets.

Fig 12 plots the resulting MAE for both light-skin and dark-skin participants at each darkness level. Performance gradually declines with increasing darkness, as predicted, but the rate of degradation varies by different skin tone variations: at 75% darkness, the MAE increases from roughly 0.61 bpm to 2.35 bpm on subjects with lighter skin tones, while on subjects with darker skin tone variations, the MAE increases from 0.95 bpm to 2.85 bpm over the same range. These findings demonstrate that our adaptive fusion strategy partially mitigates illumination challenges but also highlight the need for further improvements under extreme low-light conditions.

Table 7 provides the MAE for both skin-tone groups at three levels of darkness along with the original video. This augmented evaluation demonstrates how the model operates in a range of lighting variations and highlights both its advantages and disadvantages for real-world applications in diverse environments.

**Table 7:** MAE at different illumination levels.

Skin Tone	0%	25%	50%	75%
Light Skin	0.61	0.86	1.19	2.35
Dark Skin	0.95	1.29	1.71	2.85

Table 7 reports the MAE at varying illumination levels for both light and dark skin tones. The results highlight the impact of reduced lighting on RR estimation accuracy, with performance degradation observed under darker conditions.

## 5 Result analysis

To assess the accuracy and consistency of RR estimation across varying skin tones, we conducted detailed statistical and signal-based analyses.

**Fig 13:**
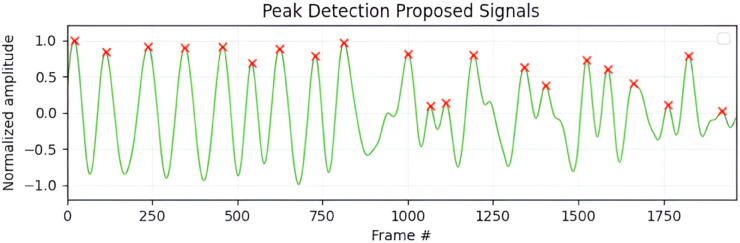
Peak detection of the processed respiratory signal. This plot illustrates the ability of the algorithm to consistently identify respiratory cycles, with clearly marked peaks corresponding to the inhalation and exhalation events in the processed waveform.

Fig 13 presents peak detection results of the proposed framework. The plot demonstrates clear identification of respiratory cycles, with distinct peaks corresponding to inhalation and exhalation events in the processed waveform, indicating reliable tracking of respiration-induced motion from facial and chest regions.

**Fig 14:**
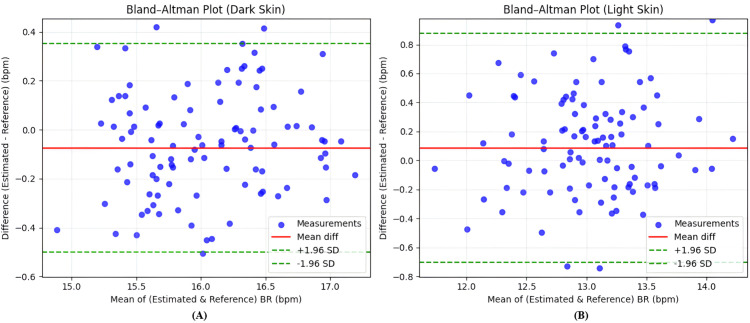
Bland–Altman plot analysis of RR estimation for different skin tones. (A) Dark skin tones exhibit a broader spread in limits of agreement, indicating greater variability and a tendency toward underestimation. (B) Light skin tones show improved agreement and reduced variability, reflecting higher estimation precision.

To evaluate the agreement between estimated and reference RR values, Bland–Altman plots are shown in Fig 14 for subjects with dark and light skin tone participants.

In Fig 14(A), dark-skinned subjects exhibit a mild underestimation trend and wider limits of agreement, indicating increased variability in RR estimation. In contrast, Fig 14(B) shows that light-skinned subjects yield narrower limits of agreement and reduced bias, reflecting more stable performance.

**Fig 15:**
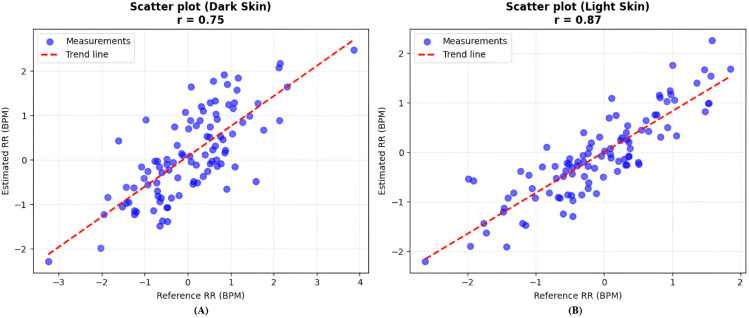
Correlation between estimated and reference RR. (A) For dark skin tones, the estimated RR shows a weaker correlation with the reference RR, with increased scatter and deviation from the identity line. (B) For light skin tones, a stronger correlation is observed, with data points more tightly clustered along the trend line.

Correlation scatter plots in Fig 15 support these observations; the model achieves a stronger linear relationship for light-skinned subjects (r=0.87) compared to dark-skinned subjects (r=0.75).

**Fig 16:**
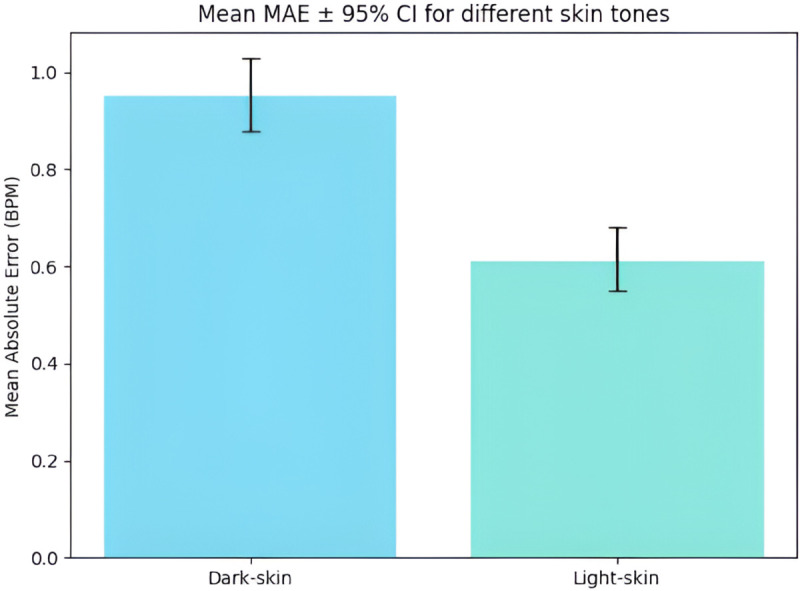
Mean MAE ± 95% CI by skin tone. Light-skin subjects show lower error and tighter confidence intervals than dark-skin subjects.

Additionally, the confidence interval (CI) bar graph in Fig 16 summarizes the mean MAE distributions for both groups. Light-skinned participants exhibit a lower average error with a narrower 95% CI, indicating improved estimation precision, while darker-skinned participants show elevated error levels and variability.

To statistically validate the observed difference in model performance between light- and dark-skinned groups, a two-tailed independent *t*-test was conducted. This test evaluates whether the observed difference in mean MAE between the two groups is statistically significant under the null hypothesis of equal group mean values.

**Fig 17:**
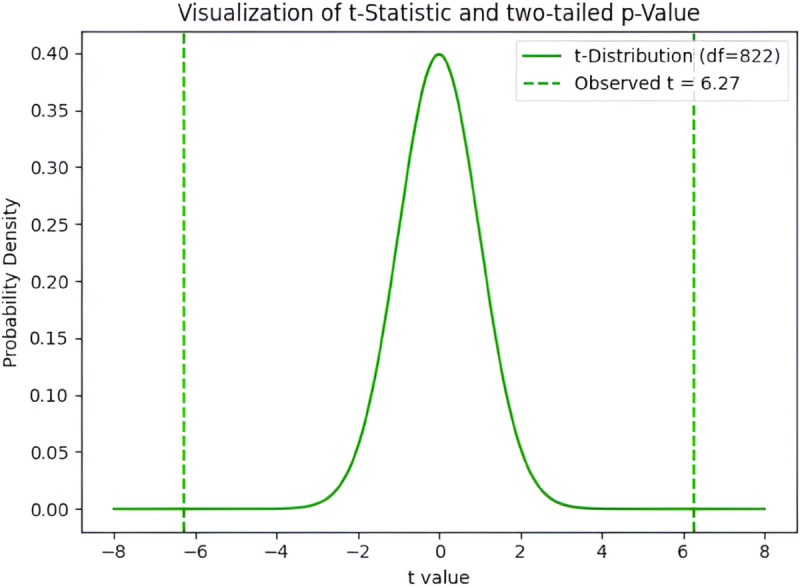
Visualization of paired t-test. The observed *t*-value (green line) lies well beyond the critical thresholds of the *t*-distribution, yielding *p* < 10^−9^.

As illustrated in Fig 17, the distribution of the t-statistic under the null hypothesis is shown alongside the observed test statistic. With degrees of freedom *df* = 822, the critical t-values for a 95% confidence level would typically lie near ±1.96. However, in our case, the observed t-statistic falls far into the extreme tails of the distribution, indicating a substantial deviation from the null hypothesis.

The resulting p-value was computed as *p* = 5.86 × 10^−10^, which is substantially lower than conventional thresholds (e.g., 0.05 or even 0.001), indicating that the difference in MAE between the two skin tone groups is not due to random variation. This result indicates a statistically significant performance difference between the two skin-tone groups.

These findings reinforce the need for robust model considerations in noncontact respiratory monitoring systems, especially when deployed in diverse populations. It also underscores the importance of evaluating fairness and generalizability across demographic subgroups.

**Fig 18:**
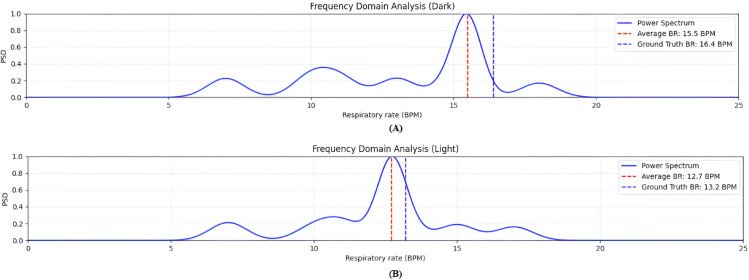
PSD analysis for dark and light Skin tone. Plot **(A)** for dark skin tones reveals a dominant respiratory frequency accompanied by multiple secondary peaks, indicating added spectral complexity—likely due to increased melanin content affecting signal extraction. In contrast, plot **(B)** for light skin tones displays a clear, isolated primary peak with minimal interference, reflecting a cleaner signal profile.

Further insights are provided through frequency-domain analysis. Fig 18 shows PSD plots of the estimated respiratory signals for dark- and light-skinned participants. For dark-skinned subjects, the PSD reveals a clear dominant peak corresponding to the RR accompanied by multiple secondary peaks, which likely contribute to increased estimation error. In contrast, the PSD for light-skinned subjects exhibits a well-isolated primary peak with minimal secondary components, indicating a cleaner signal with reduced spectral interference.

The overall result analysis confirms that the 1D CNN–spectral fusion architecture accurately estimates RR across varying contexts. In controlled environments with minimal movements, such as the PURE dataset, the model achieves minimal error, reflecting low noise levels and high signal quality. However, when applied to real-world scenarios—including ambient lighting and demographic diversity, performance becomes more susceptible to noise and signal degradation. The observed discrepancies between skin tone subgroups can be attributed to melanin concentration attenuating optical signals, reducing reflected light quality in darker skin tones. These results emphasize both the adaptability and current limitations of the system, indicating the need for further enhancements under challenging conditions.

## 6 Discussion

This work presents a fusion-based deep-learning framework that improves the robustness of contactless respiratory monitoring across varied skin tones and ambient lighting. In contrast to existing conventional approaches that often assume idealized scenarios or overlook demographic variability, the proposed architecture integrates convolutional learning with spectral signal analysis and dynamic ROI weighting. This framework enhances stability under varying environmental conditions and improves signal quality.

However, there are still some existing challenges in this study. While the system maintains high accuracy under various conditions, slight performance degradation was observed for dark-skinned subjects and in low-light environments. Additionally, all evaluations were conducted under relatively static conditions, which limits our understanding of how the system might perform in dynamic, real-world scenarios involving subject movement or fluctuating illumination.

Limited size of the dataset is another limitation. While including participants with a variety of skin tones improved demographic coverage, the total number of participants remained modest. To address this constraint and systematically explore illumination robustness, data augmentation procedures synthetically altered lighting conditions across each participant’s recordings. This approach enabled evaluation of the system’s adaptability to varied darkness levels, yielding useful insights despite the small sample size. However, future studies involving larger and more demographically representative populations are still necessary to establish broader statistical reliability and population-level validity.

From a practical deployment perspective, the proposed method offers significant advantages in both cost and complexity compared to conventional respiratory monitoring approaches. The hardware requirements consist solely of a standard RGB camera, widely available in smartphones, laptops, and consumer webcams, typically costing USD 20–50. Any RGB camera capable of recording at 30 fps with a minimum resolution of 640 × 480 pixels is sufficient for reliable operation [34]. In contrast, the Go Direct Respiration Belt used for ground truth collection costs approximately USD 125 [68], while clinical-grade respiratory monitoring equipment, such as capnography systems or medical-grade pulse oximeters, represents substantially higher costs [69].

The computational requirements of the proposed architecture remained modest. Inference time was measured at approximately 33 ms per frame on a standard consumer-grade CPU (AMD Ryzen 7 2700X) without GPU acceleration, enabling real-time processing. Compared to transformer-based [49] and 3D CNN methods [67] that typically require GPU acceleration and longer processing times, the lightweight 1D CNN design enables efficient CPU-only deployment. Contact-based systems, while computationally simpler, require sensor attachment, calibration, and periodic maintenance—factors that increase operational complexity and reduce suitability for continuous, unobtrusive monitoring [70]. The low hardware cost and minimal computational overhead make this approach well-suited for home healthcare and telemedicine settings.

In the future, this study will explore more dynamic and less constrained environments, where users may exhibit head motion, speaking, or encounter rapidly changing ambient lighting. Future work should also include clinical validation under pathological conditions such as respiratory distress, sleep apnea, and chronic pulmonary diseases. Additionally, integrating alternative modalities like radar or thermal imaging could enable robust multimodal monitoring for continuous healthcare applications.

## 7 Conclusion

This study introduced a CNN–spectral hybrid framework for noncontact RR estimation using RGB video sequences. By integrating time-domain optical flow features with classical spectral analysis and deep learning techniques, this approach leverages multiple automatically defined ROIs to enhance reliability across varying conditions. The inclusion of a spectral analysis method provides complementary temporal information and improves model stability by reducing noise artifacts and illumination variations.

Model performance on both public and custom datasets consisting of participants with skin tone variations demonstrates the effectiveness of the presented method within the tested population. Although a slight underestimation was observed in dark-skinned subjects, the model achieved low error metrics across all subgroups. These findings suggest potential adaptability to demographic differences; however, the limited sample size necessitates further validation on larger and more demographically representative groups before broader conclusions can be drawn.

The method offers a scalable, contactless solution suitable for continuous respiratory monitoring. Future studies will aim to improve motion resilience, expand demographic coverage, and explore clinical deployment in dynamic scenarios, thereby further developing accessible and equitable respiratory monitoring technologies in clinical and non-clinical application.
